# The Tribal Health System in India: Challenges in Healthcare Delivery in Comparison to the Global Healthcare Systems

**DOI:** 10.7759/cureus.39867

**Published:** 2023-06-02

**Authors:** Asitava Deb Roy, Dipmala Das, Himel Mondal

**Affiliations:** 1 Pathology, All India Institute of Medical Sciences, Deoghar, IND; 2 Microbiology, All India Institute of Medical Sciences, Deoghar, IND; 3 Physiology, All India Institute of Medical Sciences, Deoghar, IND

**Keywords:** healthcare inequality, global healthcare systems, traditional healing practices, cultural barriers, language barriers, limited infrastructure, geographical remoteness, challenges, india, tribal health

## Abstract

The tribal health system in India faces unique challenges in comparison to non-tribal health in the nation and global healthcare systems. The tribal health issues are distinct due to the diverse socio-cultural practices, rituals, customs, and languages of the tribal communities. Despite commendable efforts, there are several obstacles that hinder the successful delivery of healthcare services to these underserved populations. These challenges include geographical remoteness and limited infrastructure, language, and cultural barriers; scarcity of healthcare professionals; socioeconomic disparities; and the need for cultural sensitivity and integration of traditional healing practices. Overcoming these challenges requires collaborative efforts between the government, medical specialists, and the indigenous tribes themselves. By addressing these obstacles, it is possible to enhance the accessibility, quality, and cultural appropriateness of healthcare services for tribal groups, leading to improved health outcomes and reduced health inequalities.

## Editorial

The population of India is diverse and includes a significant number of native groups referred to as tribes or Adivasis. These tribes possess distinct socio-cultural practices, rituals, customs, and languages that set them apart from the broader civilization. It is of utmost importance for the healthcare system to address the medical needs of these underserved groups. However, despite commendable efforts, several obstacles hinder the effective provision of healthcare services to these tribal inhabitants. These challenges in healthcare delivery within these communities exhibit a unique nature when compared to challenges encountered on a national or global scale [1].

The challenges that are usually faced during health system delivery in this population are summarized below.

Geographical remoteness and limited infrastructure

Geographical remoteness and limited infrastructure pose significant challenges to the delivery of healthcare services in tribal communities in India. These communities are often located in remote and inaccessible areas, such as mountainous terrains, dense forests, or regions with inadequate transportation infrastructure. The unique geographical characteristics of these areas make it difficult to establish and maintain healthcare facilities and ensure timely and efficient delivery of medical supplies and services. The lack of nearby healthcare facilities means that individuals often have to travel long distances to receive medical attention. This can be particularly challenging for any medical emergency. Furthermore, remoteness limits healthcare service providers from reaching those areas for daycare procedures. Hence, it is challenging to deliver prompt and comprehensive healthcare services due to a lack of well-equipped hospitals, clinics, and health centers [2].

Language and cultural barriers

Language and cultural barriers pose significant challenges to healthcare delivery in tribal areas in India. These barriers impede effective communication between healthcare providers and tribal patients, leading to misunderstandings and suboptimal healthcare outcomes. Limited access to health information in native languages hinders tribal community members from making informed decisions about their health. Cultural sensitivity is crucial, as healthcare providers must understand and respect tribal customs and beliefs to establish trust and provide culturally appropriate care. Sometimes, the customs go against modern medical treatment. The lack of cultural competence among healthcare professionals further exacerbates these challenges, hindering the delivery of quality healthcare services to tribal communities. Addressing language and cultural barriers requires efforts to improve communication, provide language interpretation services, and develop culturally tailored healthcare resources. By overcoming these barriers, healthcare delivery in tribal areas can be improved, leading to better health outcomes and reduced disparities [2].

Limited access to healthcare professionals

Limited access to healthcare centers and professionals is a significant challenge in tribal health in India. Tribal communities often reside in remote and geographically inaccessible areas, which makes the government build and maintain healthcare facilities. Additionally, there is a scarcity of healthcare professionals, including doctors, nurses, and paramedical workers, in these areas. Healthcare professionals may not opt for a job in such remote places. Many state governments have started providing remote incentives to encourage young doctors to work in remote areas. However, the scarcity is still there. The inadequate availability of skilled healthcare providers creates a gap in the delivery of healthcare services, leading to delayed diagnoses, inappropriate treatment, and limited access to specialized medical care. Efforts should be made to improve transportation networks, establish healthcare facilities closer to tribal settlements, and increase the number of healthcare professionals in these regions to enhance access to quality healthcare for tribal populations in India [3].

Socioeconomic disparities

Tribal communities in India face numerous socioeconomic challenges, including widespread poverty and limited access to education. These conditions create significant barriers to accessing healthcare services and contribute to the perpetuation of health inequities. Tribal groups struggle to meet their basic needs. Hence, they may neglect medical care and treatments. As a result, many tribal individuals and families are unable to reach healthcare facilities in a timely manner. Moreover, the lack of awareness about preventive healthcare measures further exacerbates the health disparities experienced by tribal populations. Without proper education and information about disease prevention, tribal communities are more susceptible to preventable illnesses. The combination of socioeconomic challenges and limited healthcare resources creates a complex landscape in which providing equal healthcare to tribal populations becomes exceptionally challenging. Addressing these healthcare issues requires a multi-faceted approach that includes initiatives to alleviate poverty and enhance access to education related to preventive health [4].

Cultural sensitivity and traditional practices

The tribal health system in India places great importance on traditional healing practices, which are deeply rooted in the beliefs and culture of indigenous communities. Traditional healers or birth attendants play a vital role in addressing the healthcare needs of tribal populations. They possess extensive knowledge of local herbs, traditional remedies, and indigenous healing techniques. While modern medicine offers its own advantages, it is crucial to uphold and honor the cultural practices and beliefs of indigenous societies. Failing to acknowledge and integrate their indigenous healing methods may lead to distrust and reluctance among tribal populations to seek healthcare from modern facilities. Ayurveda is a traditional Indian medicine that is approved in India. This system can be of great help in tribal areas. Hence, along with modern medicine, Ayurveda and other traditional medicine system can play a vital role in delivering healthcare to tribal communities. Therefore, it is essential to foster a collaborative approach that recognizes the value of traditional healing practices while also ensuring access to and utilization of modern healthcare services. By bridging the gap between traditional and modern healthcare, it becomes possible to provide comprehensive and culturally sensitive care to tribal communities in India [5].

Thus, there are various challenges that are distinctive to the healthcare delivery in the tribal population of India when compared to the national or global healthcare systems. The government, medical specialists, and the indigenous tribes themselves must work together in a comprehensive manner to overcome these obstacles. Potential solutions are described in Table 1.

**Table 1:** Potential solutions to the current problems faced by tribal healthcare in India

Component	Brief
Enhancing healthcare infrastructure	Invest in the development and improvement of healthcare infrastructure in tribal areas, including the establishment of well-equipped hospitals, clinics, and primary healthcare centers. This will ensure that tribal communities have access to quality healthcare services closer to their settlements.
Strengthening human resources	Increase the availability of healthcare professionals, such as doctors, nurses, and paramedical workers, in tribal areas. This can be achieved through recruitment drives, incentives, and targeted training programs to attract and retain healthcare personnel in these regions.
Promoting health education and awareness	Launch health education programs that focus on preventive healthcare measures, disease management, and hygiene practices specifically tailored to the cultural and linguistic context of tribal communities. This will empower individuals to make informed decisions about their health and promote healthier lifestyles.
Enhancing outreach and mobile health services	Establish mobile healthcare units that can reach remote and inaccessible tribal areas to provide essential healthcare services, including screenings, immunizations, and basic treatments. These mobile units can bridge the gap between healthcare facilities and tribal communities, improving access to care.
Culturally sensitive healthcare	Develop culturally sensitive healthcare models that respect and incorporate traditional healing practices and beliefs of tribal communities. Collaborating with traditional healers and integrating their knowledge with modern medical practices can promote trust, engagement, and better healthcare outcomes.
Strengthening community participation	Encourage active participation of tribal communities in decision-making processes related to healthcare. Engaging community leaders, tribal representatives, and local organizations can help in designing and implementing healthcare programs that are responsive to their needs and preferences.
Government policies and support	Implement policies that prioritize tribal health and allocate sufficient funds for healthcare infrastructure, human resources, and health education programs. Government support and initiatives are crucial in addressing the unique challenges faced by tribal communities and ensuring equitable healthcare access.
Research and data collection	Conduct research and collect data specifically focused on tribal health to understand the specific health challenges, prevalent diseases, and healthcare needs of these communities. Evidence-based approaches can help in designing effective interventions and policies.

In conclusion, addressing the healthcare challenges faced by tribal communities in India requires a comprehensive and culturally sensitive approach. By investing in healthcare infrastructure, strengthening human resources, promoting health education, and enhancing outreach services, significant progress can be made in improving access to healthcare for tribal populations. Additionally, integrating traditional healing practices and beliefs, along with engaging tribal communities in decision-making processes, will foster trust and better healthcare outcomes. Government support, research, and data collection are essential for evidence-based interventions and policies that target the unique health needs of tribal communities. By implementing these potential solutions, India can work toward achieving health equity and improving the overall well-being of its tribal populations. It is imperative that all stakeholders, including government bodies, healthcare professionals, community leaders, and tribal representatives, collaborate and prioritize the health and welfare of tribal communities to ensure a brighter and healthier future for all.
